# Peroxiredoxin 6 Modulates Insulin Secretion and Beta Cell Death *via* a Mitochondrial Dynamic Network

**DOI:** 10.3389/fendo.2022.842575

**Published:** 2022-03-18

**Authors:** Francesca Pacifici, David Della-Morte, Barbara Capuani, Andrea Coppola, Maria Giovanna Scioli, Giulia Donadel, Aikaterini Andreadi, Fabiola Ciccosanti, Gian Maria Fimia, Alfonso Bellia, Augusto Orlandi, Davide Lauro

**Affiliations:** ^1^ Department of Systems Medicine, University of Rome “Tor Vergata”, Rome, Italy; ^2^ Department of Human Sciences and Quality of Life Promotion, San Raffaele University, Rome, Italy; ^3^ Department of Neurology and Evelyn F. McKnight Brain Institute, Miller School of Medicine, University of Miami, Miami, FL, United States; ^4^ Anatomic Pathology, Department of Biomedicine and Prevention, University of Rome “Tor Vergata”, Rome, Italy; ^5^ Department of Clinical Sciences and Translational Medicine, University of Rome “Tor Vergata”, Rome, Italy; ^6^ Department of Epidemiology, Preclinical Research and Advanced Diagnostics, National Institute for Infectious Diseases L. Spallanzani, Istituto Di Ricovero e Cura a Carattere Scientifico (IRCCS), Rome, Italy; ^7^ Department of Molecular Medicine, Sapienza University of Rome, Rome, Italy; ^8^ Department of Medical Sciences, Fondazione Policlinico Tor Vergata, Rome, Italy

**Keywords:** insulin secretion, oxidative stress, mitochondrial function, mitochondrial morphology, apoptosis, diabetes mellitus

## Abstract

In pancreatic beta cells, mitochondrial metabolism controls glucose-stimulated insulin secretion (GSIS) by ATP production, redox signaling, and calcium (Ca^2+^) handling. Previously, we demonstrated that knockout mice for peroxiredoxin 6 (*Prdx6^-/-^*), an antioxidant enzyme with both peroxidase and phospholipase A2 activity, develop a mild form of diabetes mellitus with a reduction in GSIS and in peripheral insulin sensitivity. However, whether the defect of GSIS present in these mice is directly modulated by Prdx6 is unknown. Therefore, the main goal of the present study was to evaluate if depletion of Prdx6 affects directly GSIS and pancreatic beta β-cell function. Murine pancreatic β-cell line (βTC6) knockdown for Prdx6 (Prdx6^KD^) was employed, and insulin secretion, ATP, and intracellular Ca^2+^ content were assessed in response to glucose stimulation. Mitochondrial morphology and function were also evaluated through electron microscopy, and by testing mitochondrial membrane potential, oxygen consumption, and mitochondrial mass. Prdx6^KD^ cells showed a significant reduction in GSIS as confirmed by decrease in both ATP release and Ca^2+^ influx. GSIS alteration was also demonstrated by a marked impairment of mitochondrial morphology and function. These latest are mainly linked to mitofusin downregulation, which are, in turn, strictly related to mitochondrial homeostasis (by regulating autophagy) and cell fate (by modulating apoptosis). Following a pro-inflammatory stimulus (typical of diabetic subjects), and in agreement with the deregulation of mitofusin steady-state levels, we also observed an enhancement in apoptotic death in Prdx6^KD^ compared to control cells. We analyzed molecular mechanisms leading to apoptosis, and we further demonstrated that Prdx6 suppression activates both intrinsic and extrinsic apoptotic pathways, ultimately leading to caspase 3 and PARP-1 activation. In conclusion, Prdx6 is the first antioxidant enzyme, in pancreatic β-cells, that by controlling mitochondrial homeostasis plays a pivotal role in GSIS modulation.

## Introduction

Diabetes mellitus is a global health problem (1) and is the seventh leading cause of death worldwide (2). It is a multifactorial chronic disease characterized by hyperglycemia, altered insulin secretion, and/or impairment in insulin action (3, 4). Type 2 diabetes mellitus (T2DM) is distinct by a massive loss in beta (β)-cell mass, which is mainly linked with apoptotic cellular death (5). Among all factors implicated in the physiopathology of T2DM, oxidative stress is pivotal in promoting β-cell dysfunction (6). Pancreatic β-cells are more susceptible compared to other tissues to oxidative stress due to their lower expression in antioxidant enzymes, such as glutathione peroxidase (Gpx), superoxide dismutase (SOD), and catalase (7).

Mitochondria, by controlling cellular respiration, are a primary source of reactive oxygen species (ROS) production and the main regulators of oxidative stress damage (8). Mitochondria, in pancreatic β-cells, also regulate glucose-stimulated insulin secretion (GSIS), by controlling ATP (adenosine triphosphate) production and intracellular calcium (Ca^2+^) influx (9). Impairment in mitochondrial physiology is, therefore, one of the major factors leading to T2DM onset (6). Recently, a central role in counteracting oxidative stress in β-cells, and therefore a potential role against T2DM, has been suggested for the peroxiredoxin antioxidant system (7). Peroxiredoxins (Prdxs) are a class of antioxidant enzymes composed of six members (10). Interestingly, among those, Prdx6 is the unique member that uses glutathione as a reducing agent, showing a bifunctional activity (phospholipase A2 and peroxidase) (10). Moreover, Prdx6 is also a key regulator of mitochondrial redox balance (11, 12) and plays a pivotal role in mitophagy/apoptosis (11). Based on the relevance of Prdx6 in regulating both oxidative stress and mitochondrial function, strictly linked to T2DM development, we previously demonstrated that knockout mice for Prdx6 (*Prdx6^-/-^*) displayed a phenotype similar to an early stage of T2DM with a reduced GSIS and a higher level of muscle insulin resistance (13). However, to the best of our knowledge, data demonstrating whether GSIS in β-cells is directly modulated by Prdx6 are still missing. Therefore, in the present study, we aimed to understand the mechanisms underlying this process by using an *in vitro* model of the Prdx6 knockdown β-cell line.

## Materials and Methods

### Cell Culture

The murine pancreatic β-cell line (βTC6) was purchased from American Type Culture Collection. Cells were cultured in Dulbecco’s modified eagle’s medium (DMEM) (Thermo Scientific, Waltham, MA, USA) supplemented with 15% fetal bovine serum (FBS) (Thermo Scientific, Waltham, MA, USA) and 100 U/ml penicillin/streptomycin (Thermo Scientific, Waltham, MA, USA). Cells were maintained at 37°C in humidified air containing 5% CO_2_.

### Stably Silenced Murine Insulinoma β-Cell Line

Hek293T cells were transfected with the lentiviral pLKO vector (containing a shRNA for Prdx6 or a shRNA for the GFP in Scramble cells) together with the Pax2 (pMDLg/p and pRSV-Rev plasmids) and ENV (VSV-G) plasmids, in order to allow viral particle assembly. The lentiviral suspension was supplemented with polybrene (4 μg/ml, Sigma-Aldrich, Saint Louis, MO, USA) and incubated with target cells (βTC6) for 8–12 hours (h). To increase transduction efficiency, infection was repeated twice. Infected cells were then analyzed starting from 48 h after the procedure.

### Insulin Secretion and Content

Insulin secretion was evaluated in Scr and Prdx6^KD^ cells by using the Mouse Insulin ELISA Kit (Mercodia, Uppsala, Sweden). Cells were plated, and 24 h later cells were starved in KREBS (135 mM NaCl, 3.6 mM KCl, 5 mM NaHCO_3_, 0.5 mM Na_2_HPO_4_, 0.5 mM MgCl_2_, 1.5 mM CaCl_2_, 10 mM HEPES, 0.1% BSA RIA grade; all reagents were ordered from Sigma-Aldrich, Saint Louis, MO, USA) for 1 h. Subsequently, cells were treated with D-glucose 30 mM (Sigma-Aldrich, Saint Louis, MO, USA) at 0, 5, 15, and 30 minutes (min). Supernatants were collected, and ELISA was performed according to the manufacturer’s protocol.

Insulin content was measured in basal conditions. Cells were permeabilized by using the Fixation/Permeabilization Solution Kit (BD) following the manufacturer’s protocol. Subsequently, cells were stained using an anti-insulin antibody (Cell Signaling, Danvers, MA, USA) and then analyzed by FACS analysis, according to Nadri et al. (14).

### ATP Production Analysis

ATP production was assessed by using the ATP Assay Kit (Abcam, Cambridge, UK) in Scr and Prdx6^KD^ cells treated as described in the previous paragraph. Following glucose stimulation, cells were lysed and centrifuged; supernatants were recovered and deproteinized following the manufacturer’s protocol. Samples were centrifuged, and supernatants were collected and added into a 96-well plate, protected from light. The OD was measured at 570 nm.

### Intracellular Ca^2+^ Assessment

Cells were treated as previously described for insulin secretion. Subsequently, Fluo4 and FuraRed (Thermo Scientific, Waltham, MA, USA) were added to each well following the manufacturer’s protocol. Next, cells were washed and then analyzed by using a fluorimeter with 494 nm excitation and 516 nm emission.

### Gene Expression Analysis

Total RNA was isolated from Scr and Prdx6^KD^ cells, by using TRIzol Reagent (Thermo Scientific, Waltham, MA, USA) as previously reported (15). Briefly, 2.5 μg of total RNA was reverse transcribed into cDNA using a High-Capacity cDNA Kit (Applied Biosystems, Foster City, CA). Subsequently, qRT-PCR was performed by using inventories under patent primers for insulin, MafA, and PDX1 (Applied Biosystems, Foster City, CA). Their relative expression was calculated using the comparative ΔΔCT method, and the values were expressed as 2^-ΔΔCT^.

### Transmission Electron Microscopy

For transmission electron microscopy (TEM) examination, basal Prdx6^KD^ and Scr cells were postfixed in 1% OsO_4_ for 2 h, dehydrated through alcohol series and propylene oxide before embedding in EPON 812 (16). Ultrathin sections were investigated with a Philips 301 transmission electron microscope. After the the acquisition, images were processed by using Adobe Photoshop C6S.

### Mitochondrial Mass and Function

Mitochondrial mass was evaluated by using MitoTracker Green Dye (Invitrogen Corp, Carlsbad, CA, USA) following the manufacturer**’**s protocol related to cytofluorimetric analysis.

To assess mitochondrial membrane potential (ΔΨM), JC1 dye was used (Cayman Chemical, Ann Arbor, MI, USA). Cells were plated, and after 24 h, JC1 Staining Solution was added. Healthy functional mitochondria showed red JC1 aggregates while unhealthy mitochondria showed green JC1 monomers. The ratio of red to green fluorescence (evaluated by cytofluorimetric analysis following the manufacturer**’**s protocol) indicated the polarization of the mitochondrial membrane.

Measurement of oxygen consumption was achieved through the phosphorescent oxygen probe MitoXpress**^®^** Xtra following the manufacturer**’**s protocol (Cayman Chemical, MI, USA). Cells were seeded into a black clear-bottom tissue culture plate. After 24 h, the growth medium was removed and replaced with a fresh medium. MitoXpress**^®^** Xtra Solution was added to each sample. The plate was read at 380 nm immediately and kinetically for 2 h.

### Western Blot Analysis

Western blot analysis was conducted as reported by Pacifici et al. (13). Briefly, cells were lysed in ice-cold buffer and then centrifuged. Supernatants were collected, and the protein concentration was determined using Bradford assay (Bio-Rad Laboratories, Milan, Italy). 50 µg of protein lysates was loaded on pre-cast 4%–12% gels (Thermo Scientific, Waltham, MA, USA), separated by SDS-PAGE, and transferred to nitrocellulose membranes using Trans-Blot Turbo™ Transfer System (Bio-Rad Laboratories, Milan, Italy). Then, antigen–antibody complexes were detected with enhanced chemiluminescence (ECL) reagent (GE Healthcare, Little Chalfont, UK) followed by exposure to ChemiDoc™ Touch Imaging System (Bio-Rad Laboratories, Milan, Italy). Bands were quantified using Image Lab™ Software (Bio-Rad Laboratories, Milan, Italy).

The primary antibodies used were Fis1 (sc-98900), Drp1 (sc-32898), Mfn1 (sc-50330), Mfn2 (sc-515647), Opa1 (sc-393296), vinculin (sc-73614), NOXA (sc-30209) (all provided by Santa Cruz Biotechnology, Santa Cruz, CA, USA), actin (3700S), caspase (9662S), PARP-1 (9542S), and LC-3 (2775S) (Cell Signaling Technology, Danvers, MA, USA).

### Apoptosis Analysis

Prdx6^KD^ and Scr cells were treated with 50 ng/ml of TNF-α as a pro-apoptotic stimulus, for 24 h. Subsequently, apoptotic sub G1 phase cells were assessed by performing propidium iodide staining followed by cytofluorimetric analysis as previously described by Rea et al. (17).

### Caspase 8 and 9 Activity

Caspase 8 and 9 activity was assessed by using colorimetric assay following the manufacturer’s protocols (Bio-Techne s.r.l., Milan, Italy). Briefly, following apoptosis induction, cells were lysed by using the specific lysis buffer supplied by the kit. Then, 100–200 μg of proteins was diluted in 50 μl of lysis buffer. Subsequently, reaction buffer (containing DTT) and the specific substrate for caspase 8 (IETD-pNA substrate) or caspase 9 (LEHD-pNA substrate) were added for 1–2 h. Samples were read at 405 nm.

### Statistical Analysis

We analyzed data using GraphPad Prism 5 (La Jolla, CA, USA). Two-way analysis of variance (ANOVA) with Bonferroni *post-hoc* test or unpaired two-tailed Student’s test, when appropriate, was determined for statistical analysis and significance. All data were expressed as mean ± SEM, as indicated. Values of p < 0.05 were considered statistically significant.

## Results

### Prdx6 Deletion Impairs Insulin Secretion by Reducing Intracellular ATP and Ca^2+^ Levels

**Figure 1:**
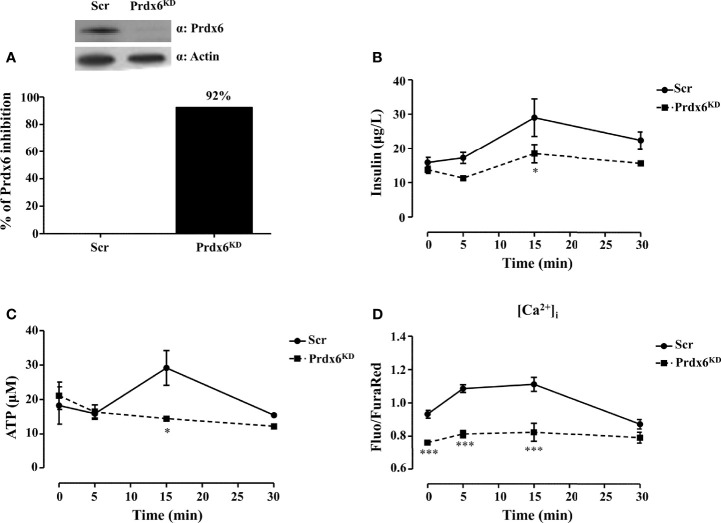
Prdx6 deletion alters insulin secretion and ATP production. **(A)** Prdx6 expression in the insulinoma βTC6 cell line stably silenced with lentiviral shRNA for Prdx6 (Prdx6^KD^, black bar) or for GFP (Scr, white bar). Steady-state levels of Prdx6 were analyzed with immunoblotting. The bar graph showed the efficiency of Prdx6 silencing reported as the percentage of inhibition of Prdx6 expression in Prdx6^KD^ cells following viral infection. **(B)** Insulin secretion was evaluated after high-glucose (30 mM) stimulation at different time points in Prdx6^KD^ (black-filled square) and Scr cells (black-filled circle). **(C)** ATP production was evaluated at different time points in Prdx6^KD^ (black-filled square) and Scr cells (black-filled circle). **(D)** Calcium content was assessed in Prdx6^KD^ (black-filled square) and Scr cells (black-filled circle) with or without glucose 30 mM. Values are expressed as mean ± SEM. *p < 0.05, ***p < 0.001 (n = 5). a.u., arbitrary units.

In a previous *in vivo* study, by using *Prdx6^-/-^* mice, we demonstrated that lack in Prdx6 resulted in insulin resistance and reduced GSIS (13). Based on these results, we sought to improve knowledge on the cellular mechanisms underlying altered insulin release in the absence of Prdx6, by using a model of pancreatic β-cell line (βTC6) knockdown for Prdx6 (Prdx6^KD^) (**Figure 1A**). Cells were stimulated with 30 mM of glucose at 0, 5, 15, and 30 min. After 15 minutes (min), according to *in vivo* results (13), Prdx6^KD^ cells showed a significant decrease in GSIS compared to control cells (Scramble, Scr) (p < 0.05) (**Figure 1B**). In the same experimental conditions, we also measured ROS, and we observed a significant increase in ROS production following glucose administration in Prdx6^KD^ compared to Scr cells (p < 0.001) (**Supplemental Figure 1**), remarking the role of ROS in impairing insulin secretion. Moreover, insulin secretion, similarly to glucose stimulation, was reduced in Prdx6^KD^ cells following KCl administration (**Supplemental Figure 2**). All these findings suggest a direct effect of Prdx6 in insulin release, independently from the stimulus.

Adenosine triphosphate (ATP) by closing ATP-sensitive potassium channels leads to voltage-gated calcium channel opening and extracellular calcium (Ca^2+^) influx which, in turn, promotes insulin release (18). A decrease in cellular ATP production, therefore, is a typical feature of β-cell dysfunction in T2DM (18). We measured intracellular levels of both ATP and Ca^2+^ using the same experimental conditions previously described. Prdx6^KD^ cells showed a significant decrease in ATP production compared to Scr (p < 0.05) following 15 min of treatment (**Figure 1C**). A significant decrease in intracellular Ca^2+^ content (**Figure 1D**) was also present not only after glucose stimulation (p < 0.001), but even at the basal state (p < 0.001). This evidence suggests an alteration in mitochondrial homeostasis and, also, in endoplasmic reticulum, as further demonstrated by the transmission electron microscopy (TEM) analysis that revealed a hypertrophied smooth endoplasmic reticulum in the absence of Prdx6 (**Supplemental Figure 3**), which may lead, in turn, to impairment in insulin release.

### Prdx6 Suppression Did Not Blunt Insulin Transcription and Synthesis

**Figure 2:**
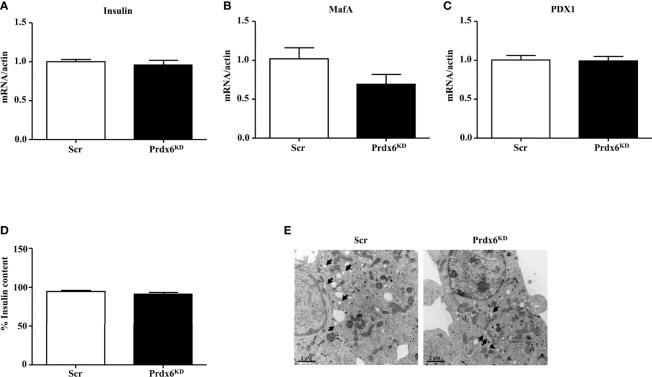
Evaluation of insulin transcription and synthesis and ultrastructural analysis of insulin granules. **(A–C)** Gene expression of Insulin, MafA, and PDX1, respectively. **(D)** Insulin content was assessed in Scr (white bar) and Prdx6^KD^ cells (black bar) by using insulin intracellular staining followed by FACS analysis. **(E)** Transmission electron images assessing granule localization in Scr and Prdx6^KD^. Original magnification: ×12,000. Values are expressed as mean ± SEM (n = 5).

Our previous *in vivo* results in isolated islets of Langerhans reported an altered secretion of insulin but not an impairment in its production (13). Then, in order to verify the hypothesis that Prdx6 modulates insulin secretion rather than its transcription, in the present study we evaluated the expression levels of insulin, V-maf musculoaponeurotic fibrosarcoma oncogene homolog A (MafA), and pancreas/duodenum homeobox protein 1 (PDX1), which are two key regulators of insulin transcription (19). As shown in **Figures 2A–C**, the expression levels of measured factors were similar between control and Prdx6^KD^ cells, suggesting that suppression of Prdx6 did not blunt insulin transcription. In agreement, also the intracellular content of insulin, evaluated by cytofluorimetric analysis according to Nadri et al. (14), was not significantly different between Scr and Prdx6^KD^ cells (**Figure 2D**), confirming our hypothesis of a Prdx6-mediated alteration in insulin secretion rather than its role on insulin transcription and synthesis.

To further validate our hypothesis, we performed an ultrastructural examination of insulin granules by using TEM analysis (**Figure 2E**). Secretion granules were evident in both cell populations, confirming that insulin was produced also in knockdown cells, according to the unaltered insulin mRNA expression. However, using TEM analysis, and in agreement with previously reported data showing decreased intracellular levels of Ca^2+^ and ATP, indicating an impairment in granules exocytosis, a different localization of insulin granules between the two groups of cells was observed. In Scr cells, in fact, granules were localized mainly near the plasma membrane, suggesting that granules were ready to be released upon an appropriate stimulus, while, in Prdx6^KD^ cells, granules were more present in the cytoplasm, thus confirming that even upon appropriate stimulation they were not readily releasable.

### Absence of Prdx6 Induces Impairment in Mitochondrial Morphology and Fate

**Figure 3:**
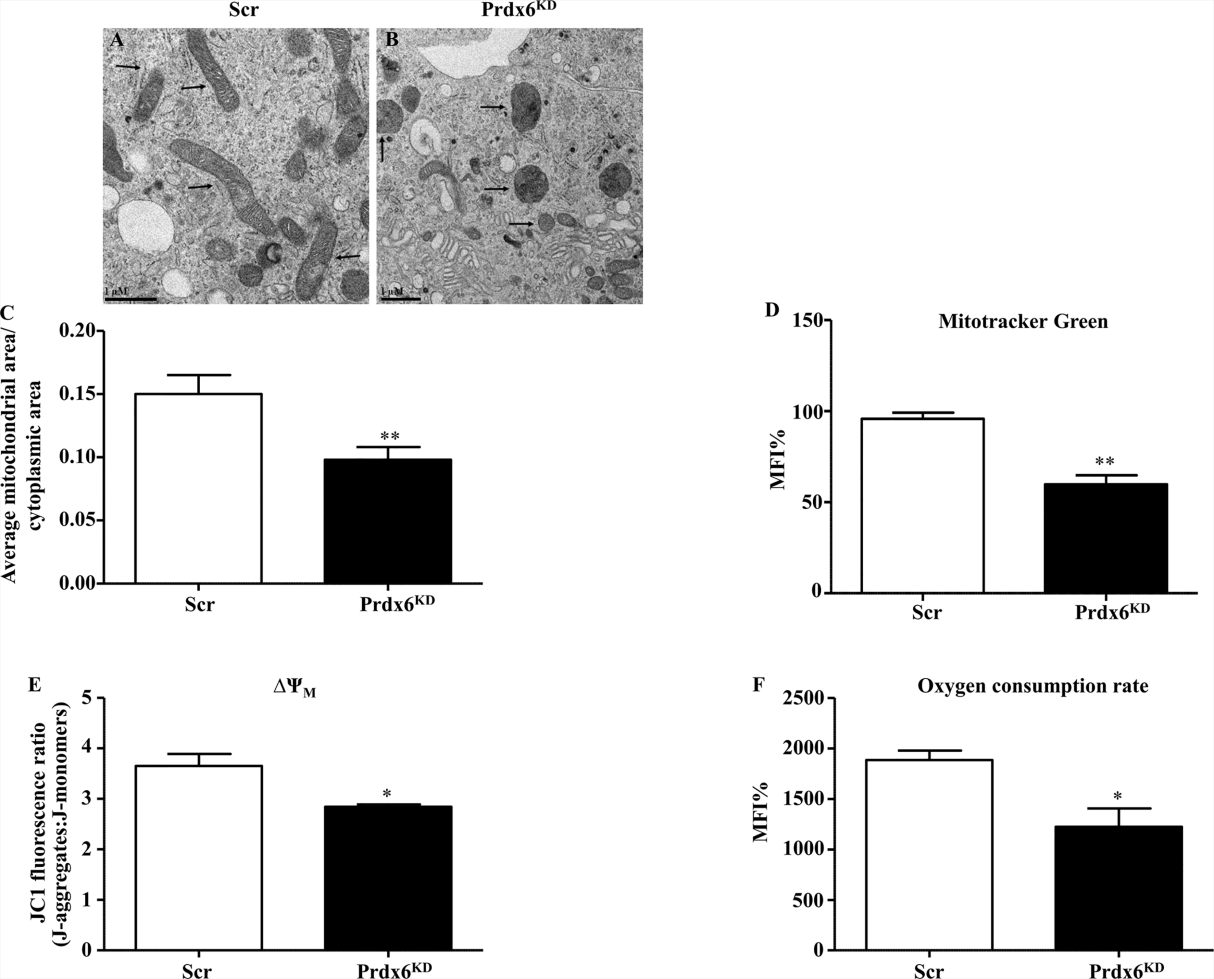
Ultrastructural and functional changes of mitochondria in β-cell knockdown for Prdx6. **(A, B)** Transmission electron images of Scr and Prdx6^KD^. Original magnification ×20,000. **(C)** The ratio between mitochondrial and cytoplasmic areas was also evaluated in both Prdx6^KD^ and Scr cell lines. **(D)** Mitochondrial mass was assessed by cytofluorimetric analysis using MitoTracker Green in both Prdx6^KD^ (black-filled bar) and Scr (white-filled bar) cell lines. **(E, F)** Mitochondrial functional state was evaluated in Prdx6^KD^ (black-filled bar) and Scr (white-filled bar) cell lines by measuring the membrane potential (ΔΨm) and oxygen consumption rate. Values are expressed as mean ± SEM. *p < 0.05, **p < 0.01 (n = 5). a.u., arbitrary units; MFI: mean fluorescence intensity.

Based on the recognized role of Prdx6 on mitochondrial homeostasis (11), and on our previous observations (13), we evaluated mitochondria morphological structure in Prdx6^KD^. TEM analysis showed that regular (Scr) cells had rounded or slightly elongated mitochondria, with internal cristae normally oriented (**Figure 3A**). Differently, in Prdx6^KD^ cells, mitochondria appeared irregular in the shape, sometimes smaller and rounded with disorganized cristae, and an anarchic arrangement (**Figure 3B**), suggesting an alteration in mitochondrial morphology.

Moreover, the ratio between mitochondrial and cytoplasmic area was determined by using TEM. As reported in **Figure 3C**, Prdx6^KD^ cells showed a significant decrease in this ratio compared to Scr cells (p = 0.015). The mitochondrial dimension was also confirmed by assessing mitochondrial volume. As shown in **Figure 3D**, Prdx6^KD^ cells displayed a decrease in mitochondrial volume, and therefore dimension, compared to Scr cells (p < 0.05), suggesting that lowering Prdx6 activity impairs both mitochondrial morphology and mass (volume). We also measured the mitochondrial network density (**Supplemental Figure 4**). No differences were observed between the two groups, confirming that lack of Prdx6 induced an alteration in mitochondrial morphology and volume rather than in their density.

To assess the functional state of mitochondria, we measured the mitochondria membrane potential (ΔΨm) and oxygen consumption rate. Both parameters were significantly lower in Prdx6^KD^ cells compared to Scr cells (p < 0.05) (**Figures 3E, F** **)**, further supporting our hypothesis that lack of cellular Prdx6 may alter mitochondrial function.

**Figure 4:**
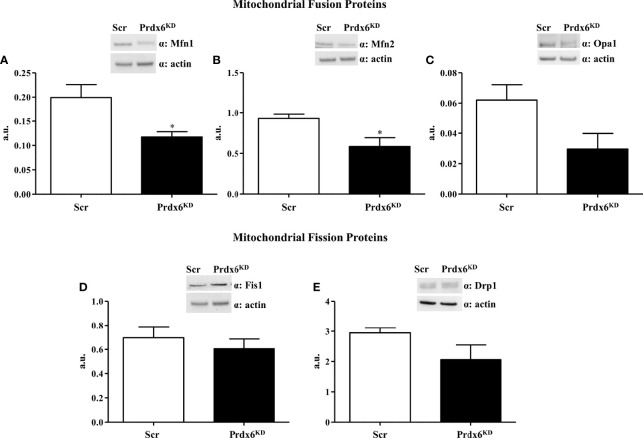
Evaluation of proteins involved in the mitochondrial dynamic network in Prdx6^KD^ cells. Fifty micrograms of Prdx6^KD^ (black-filled bar) and Scr (white-filled bar) total protein lysates were immunoblotted with specific antibodies against proteins involved in mitochondrial fusion [Mfn1 **(A)** and 2 **(B)**, Opa1 **(C)**], and fission [Fis1 **(D)** and Drp1 **(E)**]. All values were normalized with actin as a loading control. All values are expressed as mean ± SEM. *p < 0.05 (n = 5). a.u., arbitrary units.

The irregular mitochondrial shape observed in Prdx6^KD^ cells suggested a possible alteration in mitochondrial dynamics. Fission and fusion are central processes for a healthy mitochondrial network (20). Major factors controlling mitochondrial dynamics include Mitofusin 1 and 2 (Mfn1 and Mfn2) and Optic atrophy 1 (Opa1), for fusion, and dynamin-related protein 1 (Drp1) and fission protein 1 (Fis1), for fission (21). As shown in **Figures 4A, B**, Prdx6^KD^ cells had significantly lowered steady-state levels of Mfn1 and 2 compared to Scr cells (p < 0.05), confirming an altered mitochondrial dynamic network in the absence of Prdx6. However, no variation was found for Opa1, Fis1, and Drp1 (**Figures 4C–E**) Similar results were, also, observed by normalizing the protein using the mitochondrial marker ATP subunit B, as reported in **Supplemental Figure 5**.

### Mfn1 Reduction Is Mediated by the Ubiquitin-Proteasome System

**Figure 5:**
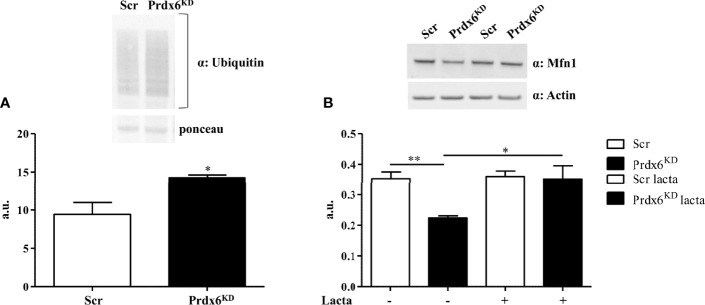
The ubiquitin–proteasome (UP) system mediates Mfn1 degradation in Prdx6^KD^ cells. Fifty micrograms of Prdx6KD (black-filled bar) and Scr (white-filled bar) total protein lysates was immunoblotted with a specific antibody against ubiquitin **(A)**, and values were normalized by using Ponceau S staining. The UP system was inhibited by lactacystin **(B)**. All values were normalized with actin as a loading control. All values are expressed as mean ± SEM. *p < 0.05, **p < 0.01 (n = 5). a.u., arbitrary units.

Following mitochondrial membrane depolarization, a ubiquitin–proteasome (UP)-dependent degradation of Mfn1 may occur (22). Since in our cellular model we observed a reduction in mitochondrial polarization in association with a decrease in Mfn1 levels, we sought to investigate whether Mfn1 reduction may be dependent on an increased activation of the UP system. First, we analyzed proteins’ ubiquitination by performing Western blot analysis. As reported in **Figure 5A**, Prdx6^KD^ cells displayed a significant enhanced protein ubiquitination compared to control cells (p < 0.01). Then, in order to evaluate whether Mfn1 reduction was UP-dependent, we treated cells with lactacystin, a well-known UP inhibitor (23). As shown in **Figure 5B**, following UP system inhibition the steady-state levels of Mfn1 were restored (p < 0.05), resulting like those of control cells. These results demonstrate that suppression of Prdx6 leads to UP activation, which in turn promotes Mfn1 degradation and alters the mitochondrial dynamic network.

### Suppression of Prdx6 Promotes Cellular Apoptosis

Reduced expression of Prdx6 increases cell death following administration of pro-inflammatory cytokine tumor necrosis factor (TNF)-α, by activating the caspase-8-mediated extrinsic apoptotic pathway (24). TNF-α sera levels are dramatically enhanced in subjects with DM, contributing to insulin resistance state (25, 26). Moreover, it contributes to the decrease in β-cell mass observed in these subjects (27–31).

**Figure 6:**
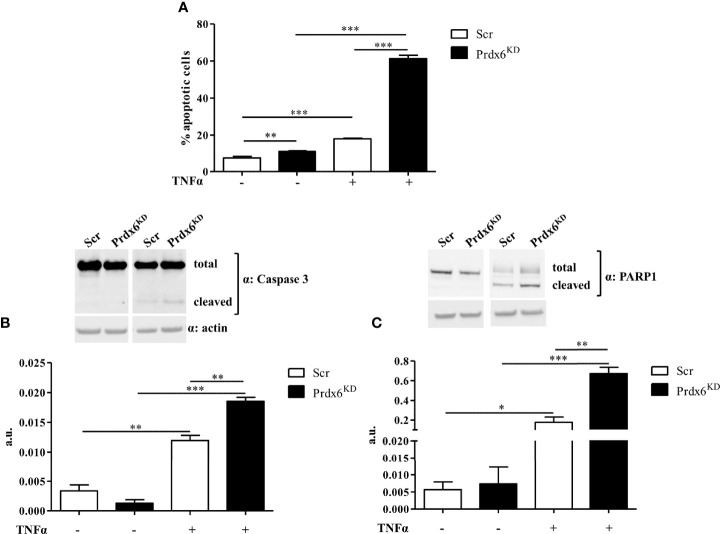
Apoptotic death induced by TNF-α. **(A)** Apoptotic cell death was analyzed by flow cytometry following propidium iodide staining in both Prdx6^KD^ (black-filled bar) and Scr (white-filled bar). Fifty micrograms of Prdx6^KD^ (black-filled bar) and Scr (white-filled bar) total protein lysates was immunoblotted with specific antibodies against caspase 3 **(B)** and PARP-1 **(C)**. All values were normalized with actin as a loading control. All values are expressed as mean ± SEM. *p < 0.05, **p < 0.01, ***p < 0.001 (n = 5). a.u., arbitrary units.

We previously reported that *Prdx6^-/-^* mice showed a significant reduction in the Langerhans islet area, suggesting a loss in β-cell mass and an increase in TNF-α expression (13). Therefore, we tested whether TNF-α administration may contribute to β-cell death in Prdx6^KD^ cells. β-Cells were treated with TNF-α 50 ng/ml for 24 h. As reported in **Figure 6A**, Prdx6^KD^ cells were more susceptible to apoptotic death induced by TNF-α compared to control cells (p < 0.001). A slight but significant increase in cell death was also observed in basal conditions in Prdx6^KD^ (p < 0.001), suggesting that lack of Prdx6 by itself predisposes to apoptosis. To further validate the TNF-α-mediated apoptotic death in our model, we assessed both caspase 3 and PARP1 cleavage, two well-established pro-apoptotic markers (**Figures 6B, C** **)** (32). In agreement with previous results, Prdx6^KD^ cells showed both increased caspase 3 (**Figure 6B**) and PARP1 (**Figure 6C**) cleavage, compared to Scr cells (p < 0.01).

**Figure 7:**
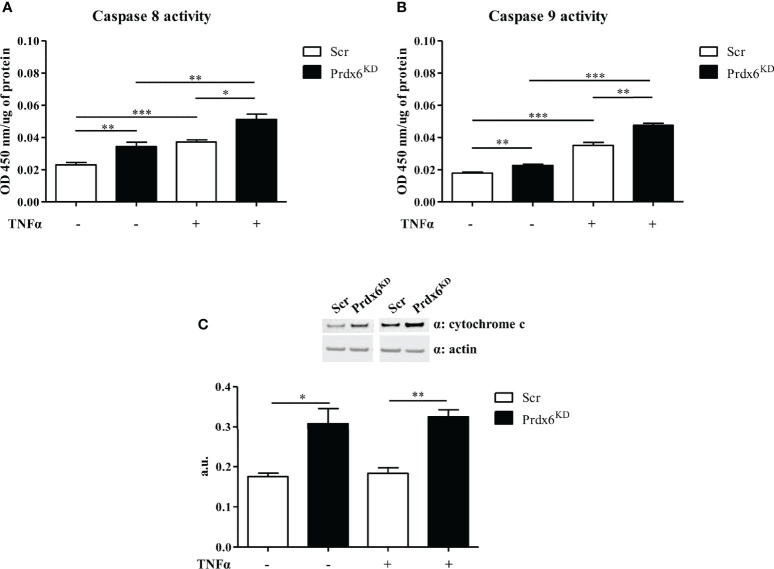
Extrinsic and intrinsic apoptotic pathways following TNF-α administration. Caspase 8 **(A)** and caspase 9 **(B)** activity was measured in both Prdx6^KD^ (black-filled bar) and Scr (white-filled bar) following treatment with TNF-α. Cytochrome c expression levels **(C)** were also analyzed in the same experimental conditions. All values were normalized with actin as a loading control. All values are expressed as mean ± SEM. *p < 0.05, **p < 0.01, ***p < 0.001 (n = 5). a.u., arbitrary units.

In order to evaluate whether TNF-α activates intrinsic or extrinsic apoptotic pathways, we evaluated the activity of both caspases 8 and 9. As shown in **Figure 7A**, TNF-α activated caspase 8 in both Prdx6^KD^ and Scr cells (p < 0.01 and p < 0.001, respectively). A slight, but significant, enhancement in caspase 8 activity was also found in basal conditions, suggesting the importance of Prdx6 in maintaining β-cell homeostasis. Interestingly, we reported that caspase 9 activity was induced by treatment with TNF-α in both cell lines (**Figure 7B**).

To further confirm the activation of the apoptotic intrinsic pathway, we analyzed the expression levels of cytochrome c, an activator of caspase 9 (33). A significant increase in cytochrome c levels was found in knockdown cells both at basal levels and after TNF-α administration, compared to control cells (p < 0.05) (**Figure 7C**). These data suggest that lack of Prdx6 promotes apoptosis by activating both intrinsic and extrinsic pathways.

## Discussion

In this study, we deeply analyzed molecular mechanisms by which Prdx6 modulates insulin release from pancreatic β-cells. Previously, by using Prdx6 knockout mouse models we demonstrated that this enzyme is crucial for glucose homeostasis, since its suppression reduced both GSIS and muscle glucose uptake (13). Here, by using the Prdx6 knockdown β-cell line, we confirmed that Prdx6 depletion impaired insulin secretion. In particular, we showed that deficiency of Prdx6 was coupled with lower intracellular levels of ATP and Ca^2+^, as well as defects on mitochondrial morphology and functionality, resulting in lower GSIS. We also reported mechanisms beyond Prdx6-mediated insulin granule secretion and Prdx6-mediated cell apoptosis. To the best of our knowledge, this is the first study investigating the cellular processes by which this antioxidant enzyme modulates glucose homeostasis. Novel potential therapeutic strategies against diabetes mellitus and metabolic diseases may benefit from these results.

ATP synthesis plays a critical role in regulating insulin secretion in β-cells *via* modulation of L-type calcium channels (18). In this study, we showed that, in addition to lower levels of ATP, Prdx6 knockdown cells also exhibited lower levels of intracellular Ca^2+^ both in basal conditions and following glucose stimulation. Variation in this pivotal electrolyte may be associated with alteration in the endoplasmic reticulum found in Prdx6^KD^ cells, since it is known that reduction in endoplasmic reticulum Ca^2+^ levels leads to a decrease in GSIS, in both human and murine islets (34).

A pivotal role for Prdx6 in mitochondrial homeostasis was also reported in a study conducted in HeLa cells, which suggested that this enzyme has a function in the clearance of damaged mitochondria (mitophagy) (11, 36). Several molecules regulate mitophagy (37). Among these, a lack of mitofusin blunts both mitophagy and autophagy, leading to an accumulation of small fragmented and damaged mitochondria (38, 39). Accordingly, here, we demonstrated that the absence of Prdx6 reduced mitofusin steady-state levels, in association with a decrease in autophagy, as reported by impairment in LC3 II activation (**Supplemental Figure 6**). These results further confirm the relevant role of Prdx6 in mitochondrial quality control and may further explain the impairment of insulin release from β-cells observed in the absence of Prdx6.

Mitochondrial fragmentation occurs before the last step of the cell life cycle, which is apoptosis (40). In particular, upon apoptosis activation, mitofusin 2 has been reported to be proteolytically degraded, leading to mitochondrial fragmentation, which in turn enhances apoptotic death (41). Accordingly, mitofusin overexpression delays apoptotic death mediated by the intrinsic pathway (42). Moreover, a decrease in mitofusin 2 levels has been demonstrated in T2DM, where beta cell apoptosis occurs, causing a reduction in beta cell mass (27, 43).

This pathological condition is characterized by chronic inflammation with enhanced levels of TNFα, which, by activating the extrinsic pathway, promotes apoptotic death in beta cells (27). Recently, it has been demonstrated that mitofusin 2 blunts hair follicle stem cell death promoted by TNFα (44). In the present study, we demonstrated that absence of Prdx6 leads to β-cell apoptosis activation mediated by both extrinsic and intrinsic pathways, in basal conditions and following pro-apoptotic inflammatory stimulus (TNFα), in association with reduced mitofusins levels.

**Figure 8:**
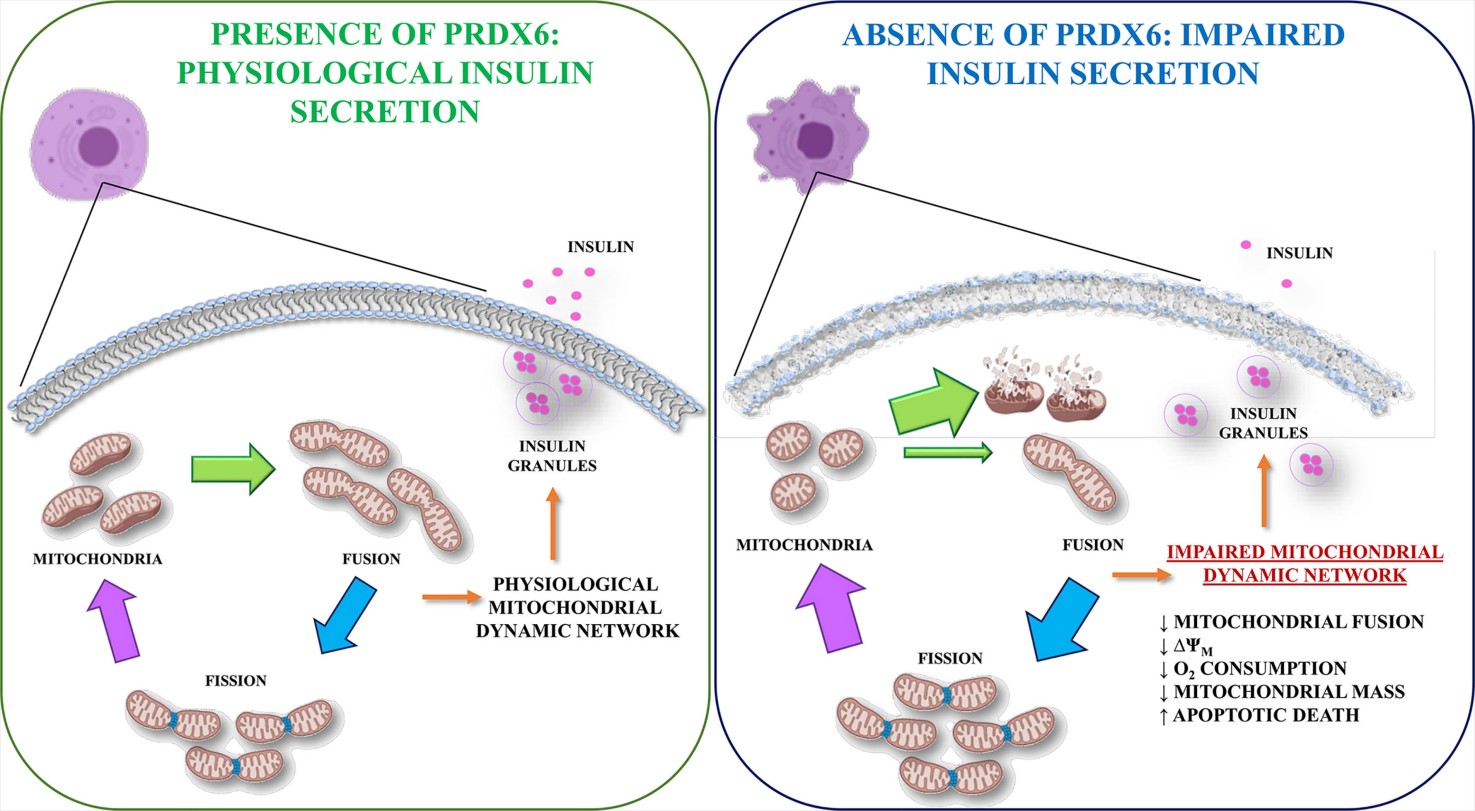
Graphical representation of Prdx6 in the regulation of GSIS. In the presence of Prdx6 mitochondria showed their physiological dynamic network leading to glucose-stimulated insulin secretion (GSIS). However, in the absence of Prx6, the mitochondrial network impairment promotes beta cell dysfunction with altered insulin secretion and beta cell apoptotic death.

Therefore, it would be possible to speculate that Prdx6, through a fine regulation of mitofusin levels, may be involved in the interplay between mitochondrial function and glucose homeostasis by triggering cellular apoptosis, explaining, at least in part, the alteration in insulin secretion and beta cell death observed in the absence of Prdx6 (**Figure 8**). In line with our results, studies demonstrating that an increase of Prdx6 protects against apoptosis are present (45).

The strength of this study includes the use of a novel Prdx6 knockdown cellular line that allowed us to better understand the underlying cellular mechanisms regulated by Prdx6. Limitations include the use of a knockdown model, which comprises a lack of the evaluation of compensatory mechanisms and difficulty in distinguishing phenotypes arising from developmental defects by those resulting from Prdx6-impaired signaling. Moreover, further limitations include the possibility to use a single dose of glucose to stimulate insulin secretion, while different doses, even lower glucose, may be helpful in clarifying the savage pathways of the cells. Further studies, possibly conducted on human beta cell lines, are needed to further confirm the pivotal role of Prdx6 in GSIS.

## Conclusion

In the present study, in the absence of Prdx6, a specific antioxidant, we showed a lower release of insulin in response to glucose stimulation. This metabolic alteration was linked with impairment in cellular mitochondrial energetic metabolism, particularly with a decrease in production of ATP and Ca^2+^. Here, we also reported that loss of mitochondrial homeostasis found in the absence of Prdx6 associated directly with unbalance in mitochondrial fission/fusion machinery. Keeping fission and fusion machinery fully functional is pivotal for cell survival as well as for maintaining mitochondrial energetic balance and β-cell mass and function. In conclusion, based on previous evidence and present results, Prdx6 may be considered a potential biomarker for diabetes mellitus and a novel therapeutic target for metabolic diseases.

## Data Availability Statement

The original contributions presented in the study are included in the article/**Supplementary Material**. Further inquiries can be directed to the corresponding author.

## Author Contributions

Conceptualization, FP, DD-M, and DL. Methodology, FP, MS, FC, GF. Validation, DD-M and DL. Formal analysis, FP, BC, AC, AA, AB, and AO. Investigation, FP. Re-sources, DL. Data curation, FP, DD-M, and DL. Writing—original draft preparation, FP and DD-M. Writing—review and editing, FP, DD-M, and DL. Visualization, FP, GD, AB, and DL. Supervision, DD-M and DL. Project administration, FP, DD-M, and DL. Funding acquisition, DL. All authors contributed to the article and approved the submitted version.

## Funding

This research was funded by the following grants: Fondazione Roma-Diabetes Mellitus, Regenerative and Reparative Processes, and Improvement of Pancreatic Beta Cell Function: Role of Bone Marrow-Mesenchymal Stem Cells, MicroRNAs, M2 Macrophages and Myeloid Derived Suppressor Cells; Fondazione Umberto Di Mario; ASI N 2013-084-R0, COREA Research Project, Italian Space Agency; The Evelyn F. McKnight Brain Institute; and PRIN 2017, #201793XZ5A_004, “Metabolic therapy of immuno-inflammation: in search for the best strategy to counteract type 2 diabetes and its complications”.

## Conflict of Interest

The authors declare that the research was conducted in the absence of any commercial or financial relationships that could be construed as a potential conflict of interest.

## Publisher’s Note

All claims expressed in this article are solely those of the authors and do not necessarily represent those of their affiliated organizations, or those of the publisher, the editors and the reviewers. Any product that may be evaluated in this article, or claim that may be made by its manufacturer, is not guaranteed or endorsed by the publisher.

## References

[1] NG F. Epidemiology of Diabetes. Medicine (2018) 47:22–7. doi: 10.1016/j.mpmed.2018.10.004

[2] WHO. Diabetes. (2020). Available at: https://www.who.int/health-topics/diabetes#tab=tab_1

[3] American Diabetes Association. Erratum. Classification and Diagnosis of Diabetes. Sec. 2. In Standards of Medical Care in Diabetes-2016. Diabetes Care (2016) 39(Suppl. 1):S13–22. doi: 10.2337/dc16-er09 27555625

[4] Lackey DE Olefsky JM. Regulation of Metabolism by the Innate Immune System. Nat Rev Endocrinol (2016) 12(1):15–28. doi: 10.1038/nrendo.2015.189 26553134

[5] Chen C Cohrs CM Stertmann J Bozsak R Speier S. Human Beta Cell Mass and Function in Diabetes: Recent Advances in Knowledge and Technologies to Understand Disease Pathogenesis. Mol Metab (2017) 6(9):943–57. doi: 10.1016/j.molmet.2017.06.019 PMC5605733 28951820

[6] Szendroedi J Phielix E Roden M. The Role of Mitochondria in Insulin Resistance and Type 2 Diabetes Mellitus. Nat Rev Endocrinol (2011) 8(2):92–103. doi: 10.1038/nrendo.2011.138 21912398

[7] Stancill JS Broniowska KA Oleson BJ Naatz A Corbett JA. Pancreatic Beta-Cells Detoxify H2O2 Through the Peroxiredoxin/Thioredoxin Antioxidant System. J Biol Chem (2019) 294(13):4843–53. doi: 10.1074/jbc.RA118.006219 PMC6442057 30659092

[8] Furukawa S Fujita T Shimabukuro M Iwaki M Yamada Y Nakajima Y. Increased Oxidative Stress in Obesity and its Impact on Metabolic Syndrome. J Clin Invest (2004) 114(12):1752–61. doi: 10.1172/JCI21625 PMC535065 15599400

[9] Wiederkehr A Wollheim CB. Impact of Mitochondrial Calcium on the Coupling of Metabolism to Insulin Secretion in the Pancreatic Beta-Cell. Cell Calcium (2008) 44(1):64–76. doi: 10.1016/j.ceca.2007.11.004 18191448

[10] Fisher AB. Peroxiredoxin 6: A Bifunctional Enzyme With Glutathione Peroxidase and Phospholipase A(2) Activities. Antioxid Redox Signal (2011) 15(3):831–44. doi: 10.1089/ars.2010.3412 PMC3125547 20919932

[11] Ma S Zhang X Zheng L Li Z Zhao X Lai W. Peroxiredoxin 6 Is a Crucial Factor in the Initial Step of Mitochondrial Clearance and Is Upstream of the PINK1-Parkin Pathway. Antioxid Redox Signal (2016) 24(9):486–501. doi: 10.1089/ars.2015.6336 26560306

[12] Lopez-Grueso MJ Lagal DJ Garcia-Jimenez AF Tarradas RM Carmona-Hidalgo B Peinado J. Knockout of PRDX6 Induces Mitochondrial Dysfunction and Cell Cycle Arrest at G2/M in HepG2 Hepatocarcinoma Cells. Redox Biol (2020) 37:101737. doi: 10.1016/j.redox.2020.101737 33035814 PMC7554216

[13] Pacifici F Arriga R Sorice GP Capuani B Scioli MG Pastore D. Peroxiredoxin 6, a Novel Player in the Pathogenesis of Diabetes. Diabetes (2014) 63(10):3210–20. doi: 10.2337/db14-0144 24947358

[14] Nadri S Barati G Mostafavi H Esmaeilzadeh A Enderami SE. Differentiation of Conjunctiva Mesenchymal Stem Cells Into Secreting Islet Beta Cells on Plasma Treated Electrospun Nanofibrous Scaffold. Artif Cells Nanomed Biotechnol (2018) 46(sup1):178–87. doi: 10.1080/21691401.2017.1416391 29241367

[15] Pacifici F Farias CLA Rea S Capuani B Feraco A Coppola A. Tyrosol May Prevent Obesity by Inhibiting Adipogenesis in 3T3-L1 Preadipocytes. Oxid Med Cell Longev (2020) 2020:4794780. doi: 10.1155/2020/4794780 33376578 PMC7746459

[16] Cervelli V Scioli MG Gentile P Doldo E Bonanno E Spagnoli LG. Platelet-Rich Plasma Greatly Potentiates Insulin-Induced Adipogenic Differentiation of Human Adipose-Derived Stem Cells Through a Serine/Threonine Kinase Akt-Dependent Mechanism and Promotes Clinical Fat Graft Maintenance. Stem Cells Transl Med (2012) 1(3):206–20. doi: 10.5966/sctm.2011-0052 PMC3659852 23197780

[17] Rea S Della-Morte D Pacifici F Capuani B Pastore D Coppola A. Insulin and Exendin-4 Reduced Mutated Huntingtin Accumulation in Neuronal Cells. Front Pharmacol (2020) 11:779. doi: 10.3389/fphar.2020.00779 32547392 PMC7270204

[18] Wang C Geng B Cui Q Guan Y Yang J. Intracellular and Extracellular Adenosine Triphosphate in Regulation of Insulin Secretion From Pancreatic Beta Cells (Beta). J Diabetes (2014) 6(2):113–9. doi: 10.1111/1753-0407.12098 24134160

[19] Zhu Y Liu Q Zhou Z Ikeda Y. PDX1, Neurogenin-3, and MAFA: Critical Transcription Regulators for Beta Cell Development and Regeneration. Stem Cell Res Ther (2017) 8(1):240. doi: 10.1186/s13287-017-0694-z 29096722 PMC5667467

[20] van der Bliek AM Shen Q Kawajiri S. Mechanisms of Mitochondrial Fission and Fusion. Cold Spring Harb Perspect Biol (2013) 5(6):a011072. doi: 10.1101/cshperspect.a011072 23732471 PMC3660830

[21] Jheng HF Tsai PJ Guo SM Kuo LH Chang CS Su IJ. Mitochondrial Fission Contributes to Mitochondrial Dysfunction and Insulin Resistance in Skeletal Muscle. Mol Cell Biol (2012) 32(2):309–19. doi: 10.1128/MCB.05603-11 PMC3255771 22083962

[22] Twig G Shirihai OS. The Interplay Between Mitochondrial Dynamics and Mitophagy. Antioxid Redox Signal (2011) 14(10):1939–51. doi: 10.1089/ars.2010.3779 PMC3078508 21128700

[23] Omura S Crump A. Lactacystin: First-in-Class Proteasome Inhibitor Still Excelling and an Exemplar for Future Antibiotic Research. J Antibiot (Tokyo) (2019) 72(4):189–201. doi: 10.1038/s41429-019-0141-8 30755736 PMC6760633

[24] Choi H Chang JW Jung YK. Peroxiredoxin 6 Interferes With TRAIL-Induced Death-Inducing Signaling Complex Formation by Binding to Death Effector Domain Caspase. Cell Death Differ (2011) 18(3):405–14. doi: 10.1038/cdd.2010.113 PMC3132003 20829884

[25] Alzamil H. Elevated Serum TNF-Alpha Is Related to Obesity in Type 2 Diabetes Mellitus and Is Associated With Glycemic Control and Insulin Resistance. J Obes (2020) 2020:5076858. doi: 10.1155/2020/5076858 32089876 PMC7013317

[26] Hotamisligil GS Peraldi P Budavari A Ellis R White MF Spiegelman BM. IRS-1-Mediated Inhibition of Insulin Receptor Tyrosine Kinase Activity in TNF-Alpha- and Obesity-Induced Insulin Resistance. Science (1996) 271(5249):665–8. doi: 10.1126/science.271.5249.665 8571133

[27] Mandrup-Poulsen T. Beta-Cell Apoptosis: Stimuli and Signaling. Diabetes (2001) 50 Suppl 1:S58–63. doi: 10.2337/diabetes.50.2007.S58 11272204

[28] Stephens LA Thomas HE Ming L Grell M Darwiche R Volodin L. Tumor Necrosis Factor-Alpha-Activated Cell Death Pathways in NIT-1 Insulinoma Cells and Primary Pancreatic Beta Cells. Endocrinology (1999) 140(7):3219–27. doi: 10.1210/endo.140.7.6873 10385418

[29] Eguchi K Manabe I. Macrophages and Islet Inflammation in Type 2 Diabetes. Diabetes Obes Metab (2013) 15 Suppl 3:152–8. doi: 10.1111/dom.12168 24003932

[30] Ishizuka N Yagui K Tokuyama Y Yamada K Suzuki Y Miyazaki J. Tumor Necrosis Factor Alpha Signaling Pathway and Apoptosis in Pancreatic Beta Cells. Metabolism (1999) 48(12):1485–92. doi: 10.1016/S0026-0495(99)90234-2 10599977

[31] Chang I Kim S Kim JY Cho N Kim YH Kim HS. Nuclear Factor kappaB Protects Pancreatic Beta-Cells From Tumor Necrosis Factor-Alpha-Mediated Apoptosis. Diabetes (2003) 52(5):1169–75. doi: 10.2337/diabetes.52.5.1169 12716748

[32] Chaitanya GV Steven AJ Babu PP. PARP-1 Cleavage Fragments: Signatures of Cell-Death Proteases in Neurodegeneration. Cell Commun Signal (2010) 8:31. doi: 10.1186/1478-811X-8-31 21176168 PMC3022541

[33] Jiang X Wang X. Cytochrome C Promotes Caspase-9 Activation by Inducing Nucleotide Binding to Apaf-1. J Biol Chem (2000) 275(40):31199–203. doi: 10.1074/jbc.C000405200 10940292

[34] Lombardi A Gambardella J Du XL Sorriento D Mauro M Iaccarino G. Sirolimus Induces Depletion of Intracellular Calcium Stores and Mitochondrial Dysfunction in Pancreatic Beta Cells. Sci Rep (2017) 7(1):15823. doi: 10.1038/s41598-017-15283-y 29158477 PMC5696524

[35] Jhun BS Lee H Jin ZG Yoon Y. Glucose Stimulation Induces Dynamic Change of Mitochondrial Morphology to Promote Insulin Secretion in the Insulinoma Cell Line INS-1e. PloS One (2013) 8(4):e60810. doi: 10.1371/journal.pone.0060810 23565276 PMC3614983

[36] Pacifici F Della Morte D Capuani B Pastore D Bellia A Sbraccia P. Peroxiredoxin6, a Multitask Antioxidant Enzyme Involved in the Pathophysiology of Chronic Noncommunicable Diseases. Antioxid Redox Signal (2019) 30(3):399–414. doi: 10.1089/ars.2017.7427 29160110

[37] Di Malta C Cinque L Settembre C. Transcriptional Regulation of Autophagy: Mechanisms and Diseases. Front Cell Dev Biol (2019) 7:114. doi: 10.3389/fcell.2019.00114 31312633 PMC6614182

[38] Song M Mihara K Chen Y Scorrano L Dorn GW 2nd. Mitochondrial Fission and Fusion Factors Reciprocally Orchestrate Mitophagic Culling in Mouse Hearts and Cultured Fibroblasts. Cell Metab (2015) 21(2):273–86. doi: 10.1016/j.cmet.2014.12.011 PMC4318753 25600785

[39] Lee WC Chiu CH Chen JB Chen CH Chang HW. Mitochondrial Fission Increases Apoptosis and Decreases Autophagy in Renal Proximal Tubular Epithelial Cells Treated With High Glucose. DNA Cell Biol (2016) 35(11):657–65. doi: 10.1089/dna.2016.3261 27420408

[40] Joaquim M Escobar-Henriques M. Role of Mitofusins and Mitophagy in Life or Death Decisions. Front Cell Dev Biol (2020) 8:572182. doi: 10.3389/fcell.2020.572182 33072754 PMC7539839

[41] Leboucher GP Tsai YC Yang M Shaw KC Zhou M Veenstra TD. Stress-Induced Phosphorylation and Proteasomal Degradation of Mitofusin 2 Facilitates Mitochondrial Fragmentation and Apoptosis. Mol Cell (2012) 47(4):547–57. doi: 10.1016/j.molcel.2012.05.041 PMC3526191 22748923

[42] Suen DF Norris KL Youle RJ. Mitochondrial Dynamics and Apoptosis. Genes Dev (2008) 22(12):1577–90. doi: 10.1101/gad.1658508 PMC2732420 18559474

[43] Rovira-Llopis S Banuls C Diaz-Morales N Hernandez-Mijares A Rocha M Victor VM. Mitochondrial Dynamics in Type 2 Diabetes: Pathophysiological Implications. Redox Biol (2017) 11:637–45. doi: 10.1016/j.redox.2017.01.013 PMC5284490 28131082

[44] Liu J Xu Y Wu Q Ding Q Fan W. Sirtuin1 Protects Hair Follicle Stem Cells From TNFalpha-Mediated Inflammatory Stress *via* Activating the MAPK-ERK-Mfn2 Pathway. Life Sci (2018) 212:213–24. doi: 10.1016/j.lfs.2018.10.003 30292830

[45] Anwar S Yanai T Sakai H. Overexpression of Peroxiredoxin 6 Protects Neoplastic Cells Against Apoptosis in Canine Haemangiosarcoma. J Comp Pathol (2016) 155(1):29–39. doi: 10.1016/j.jcpa.2016.05.002 27306414

