# Supplementary material for: Peroxiredoxin 6 Modulates Insulin Secretion and Beta Cell Death via a Mitochondrial Dynamic Network
Source: Front Endocrinol (Lausanne). 2022 Mar 18;13:842575. doi: 10.3389/fendo.2022.842575 (PMC8971298; doi:10.3389/fendo.2022.842575)
Supplement: Supplementary file 1 [file DataSheet_1.pdf]

Supplementary Material

1 Supplementary Data

1.1 ROS evaluation

ROS evaluation was performed in Scr and Prdx6^{KD} cells following glucose stimulation as reported in Material and Methods section. Then cells were treated with DCFDA 5µM for 30 min. Subsequently, cells were centrifuged and analyzed by FACS analysis.

1.2 Insulin secretion

Insulin secretion was evaluated in Scr and Prdx6KD cells by using the Mouse Insulin ELISA kit (Mercodia, Uppsala, Sweden). Cells were plated, and 24h later cells were starved in KREBS (135 mM NaCl, 3.6 mM KCl, 5 mM NaHCO3, 0.5 mM Na2HPO4, 0.5 mM MgCl2, 1.5 mM CaCl2, 10 mM HEPES, 0.1% BSA RIA grade, all reagents were ordered from Sigma Aldrich, Saint Louis, Missouri, USA) for 1h. Subsequently, cells were treated with KCl 300 µM (Sigma Aldrich, Saint Louis, Missouri, USA) for 1 h. Supernatants were collected and ELISA was performed according to the manufacturer's protocol.

1.3 Mitochondrial network density

For Transmission Electron Microscopy (TEM) examination, basal Prdx6KD and Scr cells were post-fixed in 1% OsO4 for 2h, dehydrated through alcohol series and propylene oxide before embedding in EPON 812. Ultrathin sections were investigated and photographed using a Hitachi 7100 transmission electron microscope (Tokyo, Japan). Subsequently the acquisition, cell area occupied by either mitochondria or the cytosol was outlined and measured with ImageJ software (NIH). The mitochondrial network density was quantified by two independent observers blind to the experiment. In addition, a ratio of the mitochondrial area to the cytosolic area was calculated. A total of 30 cells per sample were analyzed.

2 Supplementary Figures

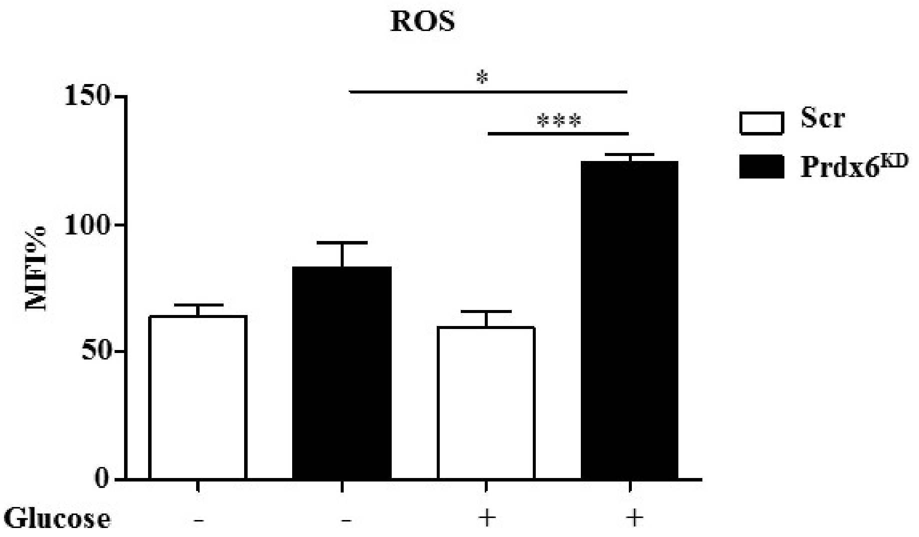

Supplementary Figure 1. ROS levels following glucose stimulation. ROS levels were evaluated in Scr (white bar) and Prdx6^{KD} cells (black bar) following glucose stimulation by using FACS analysis.

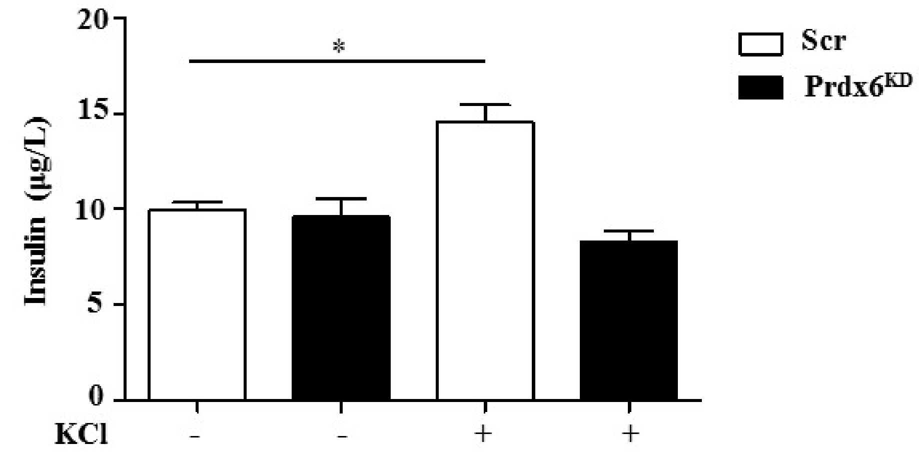

Supplementary Figure 2. Insulin content following KCl stimulation. Insulin content was assessed in Scr (white bar) and Prdx6^{KD} cells (black bar) following the stimulation with KCl 30 mM 1h. All values are expressed as mean±SEM. *$p<0.05$.

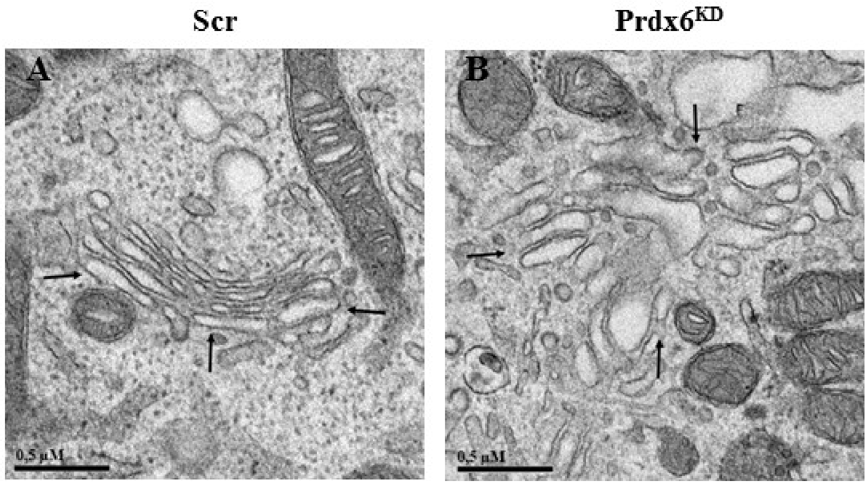

Supplementary Figure 3. Ultrastructural changes of endoplasmic reticulum in murine pancreatic β-cells lacking Prdx6. Transmission electron images of Scr (A) and Prdx6^{KD} (B) cell lines. Hypertrophied smooth endoplasmic reticulum appeared evident in Prdx6^{KD} cells compared to control cells. Original magnification: 50,000X.

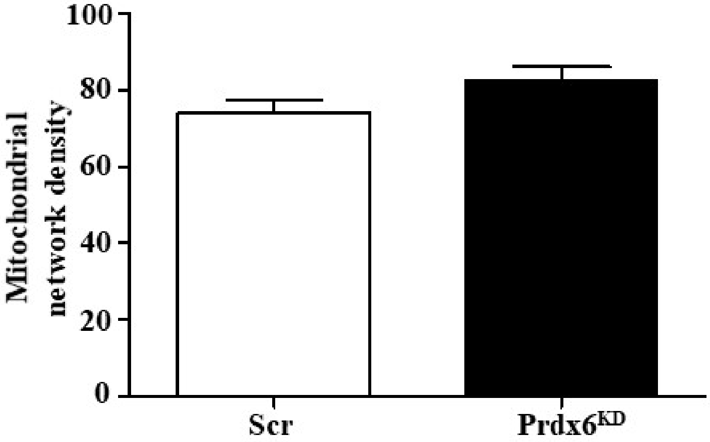

Supplementary Figure 4. Mitochondrial network density. Mitochondrial network density was assessed in Scr (white bar) and Prdx6KD cells (black bar) by using TEM examination.

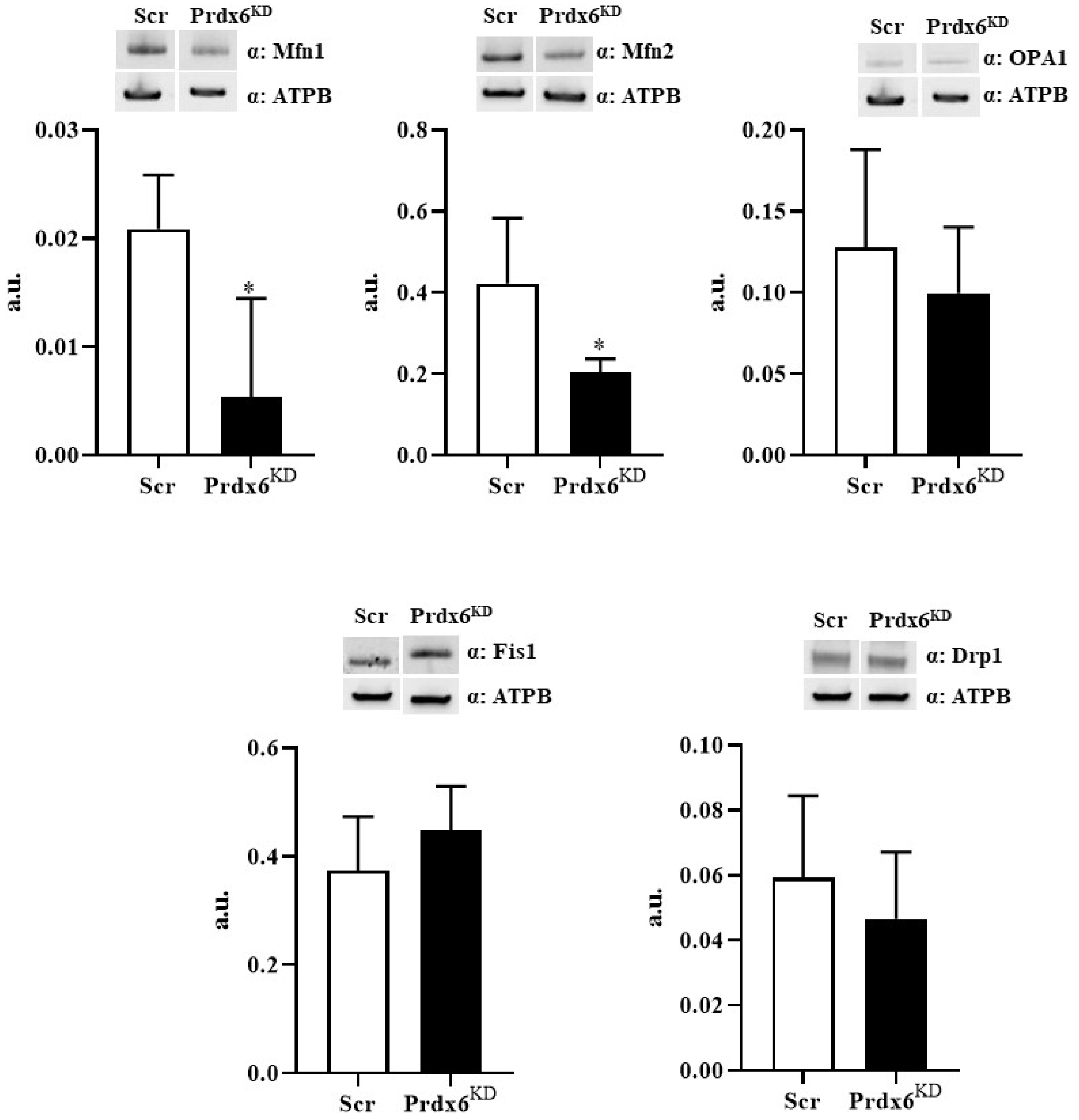

Supplementary Figure 5. Evaluation of proteins involved in the mitochondrial dynamic network in Prdx6^{KD} cells. Fifty micrograms of Prdx6KD (black filled bar) and Scr (white filled bar) total protein lysates were immunoblotted with specific antibodies against proteins involved in mitochondrial fusion (Mfn1 and 2 Opa1), and fission (Fis1 and Drp1). All values were normalized with ATP subunit B as a loading control (ATP blot is the same for Mfn1 and OPA1 since we performed the same blot and the protein have different molecular weight; similarly for Fis1 and Drp1). All values are expressed as mean±SEM. *$p<0.05$ ($n = 5$). a.u., arbitrary units.

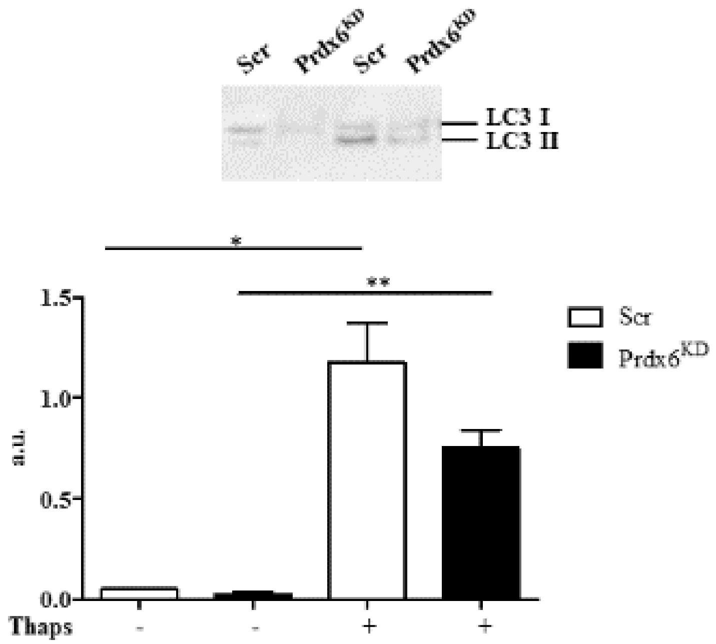

Supplementary Figure 6. LC3 I/II levels in murine pancreatic β-cells lacking Prdx6. LC3 I/II levels were evaluated in both Prdx6^{KD} (black filled bar) and Scr (white filled bar) following treatment with thapsigargin 2 μM. All values are expressed as mean±SEM. *$p<0.05$, **$p<0.01$ ($n = 5$). a.u., arbitrary units.
